# Development and preliminary validation of a machine learning system for thyroid dysfunction diagnosis based on routine laboratory tests

**DOI:** 10.1038/s43856-022-00071-1

**Published:** 2022-01-19

**Authors:** Min Hu, Chikashi Asami, Hiroshi Iwakura, Yasuyo Nakajima, Ryousuke Sema, Tsuyoshi Kikuchi, Tsuyoshi Miyata, Koji Sakamaki, Takumi Kudo, Masanobu Yamada, Takashi Akamizu, Yasubumi Sakakibara

**Affiliations:** 1 AI Strategy Team, Cosmic Corporation Co., Ltd, Tokyo, Japan; 2 First Department of Internal Medicine, Wakayama Medical University Hospital, Wakayama, Japan; 3 Department of Internal Medicine, Gunma University Hospital, Maebashi, Gunma Japan; 4 Health Control Center, Hidaka Hospital, Takasaki, Gunma Japan; 5 Present Address: Department of Internal Medicine, Kuma Hospital, Kobe, Hyogo Japan; 6 Department of Biosciences and Informatics, Keio University, Yokohama City, Kanagawa Japan

**Keywords:** Thyroid diseases, Diagnostic markers

## Abstract

**Background:**

Approximately 2.4 million patients in Japan would benefit from treatment for thyroid disease, including Graves’ disease and Hashimoto’s disease. However, only 450,000 of them are receiving treatment, and many patients with thyroid dysfunction remain largely overlooked. In this retrospective study, we aimed to develop and conduct preliminary testing on a machine learning method for screening patients with hyperthyroidism and hypothyroidism who would benefit from prompt medical treatment.

**Methods:**

We collected electronic medical records and medical checkup data from four hospitals in Japan. We applied four machine learning algorithms to construct classification models to distinguish patients with hyperthyroidism and hypothyroidism from control subjects using routine laboratory tests. Performance evaluation metrics such as sensitivity, specificity, and the area under receiver operating characteristic (AUROC) were obtained. Techniques such as feature importance were further applied to understand the contribution of each feature to the machine learning output.

**Results:**

The results of cross-validation and external evaluation indicated that we achieved high classification accuracies (AUROC = 93.8% for hyperthyroidism model and AUROC = 90.9% for hypothyroidism model). Serum creatinine (S-Cr), mean corpuscular volume (MCV), and total cholesterol were the three features that were most strongly correlated with the hyperthyroidism model, and S-Cr, lactic acid dehydrogenase (LDH), and total cholesterol were correlated with the hypothyroidism model.

**Conclusions:**

We demonstrated the potential of machine learning approaches for diagnosing the presence of thyroid dysfunction from routine laboratory tests. Further validation, including prospective clinical studies, is necessary prior to application of our method in the clinic.

## Plain language summary

Thyroid dysfunction, including over-activity of the thyroid (hyperthyroidism) and under-activity (hypothyroidism), is a leading hormonal disorder with major health implications, and many patients with thyroid dysfunction are not formally diagnosed. We aimed to develop a computer-based approach for screening patients for hyperthyroidism and hypothyroidism who might benefit from medical treatment. We used routine laboratory data and specialised computer software to detect thyroid dysfunction in patients. We show that we can do this accurately, and so might be able to use our system to screen patients based on data from routine medical tests. Further testing in clinical studies is needed, however, before our approach can be used in the clinic.

## Abstract (web summary)

Hu et al. develop a machine learning approach for diagnosis of thyroid disorders based on routine clinical laboratory measurements. The approach is tested on data from multiple institutions and can distinguish patients with hyper- or hypothyroidism from controls with high accuracy.

## Introduction

Thyroid dysfunction is a leading endocrine disorder with major health implications, including an increased risk of heart disease and hypercholesterolemia. One of the greatest challenges in thyroid dysfunction treatment is not to overlook or misdiagnose these diseases. Thyroid hormone excess and deficiency are frequently misunderstood and are too often overlooked and misdiagnosed^1^. For hyperthyroidism, the diagnosis may be delayed or missed because some symptoms can be easily attributed to other conditions, such as stress^2^, and are often mistaken for cardiac disease or gastrointestinal malignancies. Hypothyroidism can present with nonspecific constitutional and neuropsychiatric complaints, and patients with hypothyroidism are often misdiagnosed with dementia, cardiac disease, liver disease, or hyperlipidemia and therefore do not receive proper treatment^3^. The American Association of Clinical Endocrinologists has estimated that in the United States, ~4.78% of the population has misdiagnosed thyroid dysfunction^4^, and the authors argue that ~15 million adults are estimated to have unrecognized thyroid disease^5^. In Japan, it is estimated that ~2.4 million patients need treatment for thyroid disease^6^. However, only ~450,000 of them are receiving treatment. Thus, patients with thyroid dysfunction are frequently overlooked and misdiagnosed^6^.

Hyperthyroidism is a condition that occurs due to excessive production of thyroid hormones. The first step to diagnose hyperthyroidism is to measure thyroid-stimulating hormone (TSH), free thyroxine (FT4) and free triiodothyronine (FT3)^7^. In contrast, hypothyroidism is a condition in which serum thyroid hormones are decreased. Typical diseases involving hypothyroidism include Hashimoto’s disease and are diagnosed by anti-thyroid antibody tests such as anti-thyroid peroxidase antibody (TPO) and anti-thyroglobulin antibody (TgAb)^7^. Despite their clinical significance, thyroid function tests and anti-thyroid antibody tests are not included in Japanese national health checkups.

As a popular and effective approach to predictive analytics, machine learning is highly regarded due to its success in diagnosis, prediction, and choice of treatment. Recently, an emerging technique in the field of medical informatics has employed machine learning to accurately derive insights from medical records to support clinical screening and to predict disease misdiagnosis^8^. For instance, a study emphasized the superiority of machine-learning technology for predicting cardiovascular risk from routine clinical data^9^. In another study, the incidence of myocardial infarction or cerebral infarction was predicted using the results of a health checkup^10^. Numerous studies have also attempted to assess the efficacy of detecting misdiagnosed diseases, including thyroid dysfunction^11–17^. Aoki et al.^16,17^ found that there were strong, multiple correlations between a set of routine clinical parameters and FT4 in patients with both overt hyperthyroidism and overt hypothyroidism. These studies used pattern recognition methods such as neural networks and predicted the likelihood of thyroid dysfunction from a set of routine clinical tests.

Despite such efforts, there are still several concerns regarding machine-learning applications in the diagnosis of disease. These include the issues of data cleaning, missing value completion, dysfunction labeling criteria, the integration of multiple hospital datasets, and the validation and interpretation of machine-learning models. In this study, we developed an explainable diagnosis support system using machine-learning algorithms to identify thyroid dysfunction with routine clinical data, and demonstrated the potential to improve medical screening and prevent overlooking and misdiagnosing thyroid dysfunction. High accuracy was achieved in the discrimination of evident hyperthyroidism and hypothyroidism using 23 routine laboratory tests, and these features can be useful for individuals who are not thyroid disease specialists.

## Methods

### Data source

In the present study, we acquired laboratory finding datasets from different clinical university medical institutions in Japan, including Wakayama Medical University Hospital, Gunma University Hospital, Hidaka Hospital, and Kuma Hospital. The anonymized EMRs included age, sex, diagnosis codes for insurance billing, prescribed drugs, and biochemical test results.

A sample of 176,727 subjects aged 13 to 88 from different regions in Japan between 2004 and 2019 were included in our study, as illustrated in Table 1. Among the four institutions, Wakayama Medical University Hospital and Gunma University Hospital are hospitals affiliated with a medical college, Hidaka Hospital is a regional medical care support hospital, and Kuma Hospital is a hospital specializing in thyroid diseases. The data of the 176,727 subjects consisted of physician evaluations, prescriptions, clinical examinations, and laboratory findings. The physician evaluations addressed medical history, medication use, and differential diagnosis, among other topics. If a subject was prescribed medication, the name and dose of the prescription were recorded. The examinations involved anthropometric measurements and laboratory tests, among others. The institutional ethics review boards of the four institutions at which the study was conducted gave their approval (Approval Numbers: Wakayama Medical University Hospital: 2301, Hidaka Hospital: 257, Gunma University Hospital: HS2018-245, Kuma Hospital: 20180208-4). All methods were performed in accordance with the relevant guidelines and regulations, including ethical guidelines for Medical and Health Research Involving Human Subjects presented by the Ministry of Health, Labor and Welfare in Japan. According to the ethical guidelines for Medical and Health Research Involving Human Subjects, with this study design, written informed consent is not required, but we widely disclosed the outline of our study and provided opportunities for unenrollment.

**Table 1:** Summary of the data from each institution.

Institution	Wakayama Medical University	Gunma University	Hidaka Hospital	Kuma Hospital
Number of prescriptions	8,249,286	34,561,268	23,450	61,590
Number of patients	14,249	27,133	10,482	124,863
Average age	60.9	51.7	47.7	50.3
Male/female ratio	1.03 (5,888/5,723)	0.53 (8,143/15,296)	1.82 (15,125/8,325)	0.21
Data period	2010–2018	2004–2019	2004–2007	2007–2020

The demographic summary is shown for each institution. “Number of prescriptions” represents the number of prescription records in each dataset, and “Number of patients” represents the number of patients in each dataset. “Average age”, and “male/female ratio” and “data period” represent the demographic summary of the patients in each institution.

The K-nearest neighbor (KNN) algorithm was used to predict and complement the missing values, with k set to 3 in the data filling process. A previous study^11^ reported the KNN algorithm to substantially increase the number of applicable subjects. Compared with missing value deletion, the KNN algorithm is easily applied, performs well for nonparametric datasets, and provides a larger sample size. Furthermore, since the age and sex distributions were different among the institutions, as shown in Table 1, we also conducted random undersampling to address these differences. From this dataset, the model was constructed using thyroid patient data from Wakayama Medical University and Gunma University and control group data from Hidaka Hospital and was evaluated using cross-validation. To validate the external data, the model was also evaluated on the dataset from Kuma Hospital.

### Construction of the machine-learning model

As shown in Table 2, four verification items were devised in this study to improve the performance of our machine-learning model. The criteria of data labeling and the combination of multiple institutions were evaluated first. Then, four different machine-learning algorithms and three sets of input features were evaluated to achieve the best performance of our thyroid dysfunction classification models.

**Table 2:** List of verification items.

No.	Verification item	Option			
1	Training data labeling	Thyroid function test criterion	Prescription criterion		
2	Institution combination (for patient data and control group data)	Institution combination 1 (Inst. comb. 1)	Institution combination 2 (Inst. comb. 2)	Institution combination 3 (Inst. comb. 3)	External
3	Machine-learning algorithm	GBDT	SVM	Logistic regression	ANN
4	Input features	Feature set 1	Feature set 2		

Verification items in this study are categorized into four groups: “Training data labeling”, “Institution combination”, “Machine-learning algorithm”, and “Input features”. Each category contains several specific verification options and was verified in our experiments.

### Data labeling criterion

According to the guidelines of the Japanese Thyroid Association for the diagnosis of hyperthyroidism and hypothyroidism, if thyroid disorder is suspected from the clinical findings, first, a thyroid function test (TSH and FT4 measurement) is conducted, and on the basis of these results, thyroid disorder is classified into three categories—hyperthyroidism, hypothyroidism, or euthyroidism^7^. Therefore, we devised and compared the performance of two data labeling criteria.

We first devised the labeling criterion by using the result of the thyroid function test as a reference (hereinafter referred to as the “thyroid function test criterion”). Specifically, in the dataset from Wakayama Medical University, FT4 and TSH were measured with ECLusys kits (Roche Diagnostics GmbH, Mannheim, Germany). TSH < 0.5 and FT4 > 1.7 were defined as overt hyperthyroidism, and TSH > 5.0 and FT4 < 0.9 were defined as overt hypothyroidism (TSH unit: μIU/mL; FT4 unit: ng/dL). In the dataset from Gunma University, in which FT4 and TSH were measured with the Architect kits, TSH < 0.35 and FT4 > 1.48 were defined as overt hyperthyroidism, and TSH > 4.94 and FT4 < 0.7 were defined as overt hypothyroidism.

Data for the control group were extracted from the third institution, Hidaka Hospital, and consisted of the test results from regular medical examinations. We extracted comprehensive medical examination data for subjects who did not have any symptoms, suggesting thyroid dysfunction or abnormal values in the laboratory tests of TSH and FT4. The normal ranges were set to 0.34–3.88 μIU/mL for TSH and 0.95–1.74 ng/dL for FT4. Random undersampling was conducted for the control group in such a way that the sample size of the control group was equivalent to the sizes of the hyperthyroidism and hypothyroidism groups.

The thyroid function test criterion required both TSH and FT4 test results, but a small number of patient records had both of these results. Therefore, as an alternative solution, we devised another criterion of labeling the training data according to the presence of a prescription for thyroid disorder (hereinafter referred to as the “prescription criterion”). Specifically, the use of the prescription criterion satisfies the following conditions: (a) it includes patient records with standard prescribed medications for thyroid dysfunction (including thiamazole, propylthiouracil, and potassium iodide for the hyperthyroidism group and levothyroxine and thyronamine for the hypothyroidism group) obtained at the patients’ first visits, (b) it includes patients not diagnosed with thyroid nodules, (c) it includes patient records containing laboratory findings obtained within four weeks after the patient’s first prescription, and (d) it excludes records with missing values for more than half of our selected features. Since the age distributions were different among the institutions, as shown in Table 1, we also conducted data sampling to address these differences. Between the two criteria designed in this study, we focused on evaluating thyroid function test criteria as the gold standard criteria while exploring the effect of the prescription criteria, which may benefit from a larger dataset.

In machine learning, a control group is generally used as a negative label. Since hyperthyroidism and hypothyroidism are conditions of thyroid dysfunction, both often express similar symptoms and effects on some routine laboratory findings (e.g., Hb is decreased in both hyperthyroidism and hypothyroidism patients). Therefore, we considered the confounding of hyperthyroidism and hypothyroidism as “crosstalk” and refined the labeling criteria in such a way that the negative label was set as both the healthy subjects of the control group and the patients with the opposite type of thyroid dysfunction. For instance, in the data labeling process of the hyperthyroidism classification model, the hyperthyroidism group was set as a positive label, whereas both healthy subjects in the control group and hypothyroidism patients were set as a negative label.

### Integrating multiple hospital datasets

The demographics were different among the three institutions in different districts. To investigate the effect of integrating the datasets from these three hospitals, we explored three combinations of datasets to increase the generalizability of our models. Specifically, three dataset options, namely, thyroid dysfunction group data from both Wakayama Medical University and Gunma University and control group data from Hidaka Hospital (referred to as Inst. comb. 1), thyroid dysfunction group data from Wakayama Medical University and control group data from Hidaka Hospital (referred to as Inst. comb. 2), and thyroid dysfunction group data from Gunma University and control group data from Hidaka Hospital (referred to as Inst. comb. 3), were set to train and evaluate the models.

### Machine-learning algorithms

Four representative machine-learning algorithms were applied, and their thyroid dysfunction classification performance was evaluated:

The gradient boosting decision-tree (GBDT), as proposed by Friedman^18^, produces a prediction model in the form of an ensemble of weak prediction models, typically decision trees. The GBDT is based on a machine-learning technique that consists of an “ensemble” family of algorithms, creates multiple models (called weak learners), and combines them to increase prediction accuracy. The main idea of this technique is to build a set of decision trees and use them to classify a new case. Each decision-tree is generated using randomly selected variable subsets from all feature variables and a randomly selected subset of data combined by bootstrapping^19^. In this study, we employed the most accurate algorithm, called CATBoost^20^, in the GBDT family.

The artificial neural network (ANN) is a well-established classification technique that is widely used in pattern recognition studies. In general, an ANN consists of 3 layers: an input layer that receives information, a hidden layer that processes information, and an output layer that calculates the results^21^. In the present study, a standard feed-forward ANN was applied due to its relative simplicity and stability.

The support vector machine (SVM) is a supervised machine-learning technique that is widely used in pattern recognition and classification problems. In this method, each data sample is a vector whose dimensions are equal to the number of features to be considered, and the SVM creates a hyperplane that separates samples into two categories. The induced hyperplane is constructed to maximize its distance from the samples of both classes. This algorithm achieves high classification performance by using special nonlinear functions called kernels to transform the input space into a multidimensional space^22^. In this study, the radial basis function kernel was used.

Logistic regression is a statistical classifier that provides the probability of predicting the labeled class of categorical type by using a number of attributes. Logistic regression is frequently used to examine the risk relationship between disease and exposure, with the ability to test for statistical interaction and control for multivariable confirmation^23^. Logistic regression is a linear model and is used as the baseline model for the performance comparison.

### Explanatory features (variables) for machine learning

In terms of the input feature used in machine-learning models, we used the following 23 features referred to as Feature set 1, which are all features available at the four hospitals: sex, aspartate aminotransferase (AST), alanine aminotransferase (ALT), gamma-glutamyl transpeptidase (γ-GTP), red blood cell count (RBC), serum creatinine (S-Cr), alkaline phosphatase (ALP), uric acid (UA), lactic acid dehydrogenase (LDH), total protein (TP), blood urea nitrogen (BUN), albumin, albumin/globulin ratio (A/G), total cholesterol, total bilirubin (TB), C-reactivate protein, white blood count (WBC), hemoglobin (Hb), platelet, hematocrit, mean corpuscular volume (MCV), mean corpuscular hemoglobin (MCH), and mean corpuscular hemoglobin concentration (MCHC). To further verify the performance of the model depending on the set of most basic laboratory tests for our aim of rapid screening for overlooked patients, we trained and evaluated a model limited to five routine tests referred to as Feature set 2, including AST, ALT, γ-GTP, total cholesterol, and sex, of which four features are the required (mandatory) laboratory tests conducted in the Japanese national health screening program called Specific Health Checkups.

### Model validation

Cross-validation was applied to evaluate the performance of our machine-learning method in classifying patients. The evaluation was conducted by extracting 9/10 training data and 1/10 test data by conducting 10-fold cross-validation. This was repeated 10 times to extract the training and test data uniformly, and the average and standard deviation of each evaluation score of each time were calculated. During the model training and test process, we avoided including the same subject in both the training dataset and test dataset. The following measures were used for the performance evaluation criteria: the area under the receiver operating characteristic curve (AUROC); positive predictive value (PPV), defined as TP/(TP + FP); negative predictive value (NPV), defined as TN/(TN + FN); sensitivity, defined as TP/(TP + FN); and specificity, defined as TN/(TN + FP), where TP is the number of true positives, TN is the number of true negatives, FP is the number of false positives, and FN is the number of false negatives. Note that the cutoff value for classification as positive or negative is determined by the Youden^24^ index.

In addition, the data of Kuma Hospital were employed for external validation. The model was constructed using the hyperthyroidism group and the hypothyroidism group of Wakayama Medical University and Gunma University and the control group of Hidaka Hospital as the training data. The model was evaluated using the hyperthyroidism group and hypothyroidism group of Kuma Hospital and the control group of Hidaka Hospital (referred to as External).

### Feature importance

To further understand how each feature contributes to the classification of patients in our model, we introduced feature importance. Feature importance represents the factor by which the model error is increased compared to the original model error. In the decision-tree-based machine-learning algorithms, including the GBDT, impurities and the features at which the node is split are recorded for all the nodes when the decision-tree-learning process is finished, and the decision-tree calculates the feature importance using this information^19^.

### Reporting summary

Further information on research design is available in the Nature Research Reporting Summary linked to this article.

## Results

### Model validation

Table 3 summarizes the performance results of the machine-learning model constructed in this study. According to the result of 10-fold cross-validation, as shown in No. I of Table 3, the best classification model for overt hyperthyroidism achieved an AUROC of 93.8%, sensitivity of 89.1%, and specificity of 88.6%. The best classification model for overt hypothyroidism achieved an AUROC of 90.9%, sensitivity of 82.4%, and specificity of 86.5%. In the external evaluation, as shown in No. IX of Table 3, the classification model for overt hyperthyroidism achieved an AUROC of 97.2%, and the classification model for overt hypothyroidism achieved an AUROC of 94.0%.

**Table 3:** Results of the validation of models with different labeling criteria, machine-learning algorithms, institutions, and input features.

No.		I	II	III	IV	V	VI	VII	VIII	IX
Training	Data labeling	Thyroid function test criterion	Prescription criterion	Thyroid function test criterion						
	Institution combination	Inst. comb. 1	Inst. comb. 1	Inst. comb. 2	Inst. comb. 3	Inst. comb. 1				
	Machine-learning algorithm	GBDT				SVM	Logistics regression	ANN	GBDT	
	Input features	Feature set 1							Feature set 2	Feature set 1
Validation	Labeling criteria	Thyroid function test criterion								
	Institution combination	Inst. comb. 1								External
Hyperthyroidism	AUROC	93.8 ± 2.7%	93.0 ± 2.3%	93.1 ± 2.4%	92.8 ± 3.0%	91.8 ± 3.4%	90.9 ± 3.3%	91.9 ± 2.7%	85.5 ± 3.9%	97.2 ± 0.5%
	PPV	80.3 ± 6.2%	81.4 ± 4.7%	76.6 ± 8.1%	78.2 ± 7.1%	71.6 ± 4.5%	79.4 ± 6.8%	73.9 ± 7.9%	72.4 ± 7.0%	98.5 ± 0.5%
	NPV	94.4 ± 2.7%	93.1 ± 3.4%	93.9 ± 2.7%	92.4 ± 3.5%	94.1 ± 3.7%	91.7 ± 3.7%	93.6 ± 3.4%	88.5 ± 4.2%	67.4 ± 6.0%
	Sensitivity	89.1 ± 5.8%	86.4 ± 7.7%	88.6 ± 5.5%	85.4 ± 7.0%	89.4 ± 6.7%	83.6 ± 8.4%	88.3 ± 7.0%	77.3 ± 9.7%	90.0 ± 2.9%
	Specificity	88.6 ± 4.7%	89.9 ± 3.5%	85.7 ± 6.3%	87.7 ± 5.1%	82.0 ± 4.1%	88.7 ± 4.6%	83.6 ± 6.7%	84.6 ± 5.9%	93.7 ± 2.1%
Hypothyroidism	AUROC	90.9 ± 3.3%	92.1 ± 3.2%	89.3 ± 2.2%	88.5 ± 4.5%	88.6 ± 4.0%	86.7 ± 3.1%	89.0 ± 3.6%	82.5 ± 3.7%	94.0 ± 1.5%
	PPV	79.9 ± 8.4%	73.9 ± 6.2%	73.2 ± 7.4%	72.9 ± 8.1%	74.1 ± 7.0%	67.7 ± 6.6%	71.6 ± 9.2%	70.0 ± 10.3%	59.8 ± 5.2%
	NPV	91.3 ± 5.3%	94.8 ± 3.8%	92.3 ± 4.7%	91.7 ± 3.6%	90.1 ± 2.3%	90.4 ± 4.3%	92.5 ± 2.9%	85.2 ± 2.5%	98.3 ± 0.8%
	Sensitivity	82.4 ± 12.5%	90.5 ± 7.2%	85.1 ± 10.2%	84.9 ± 6.7%	81.2 ± 4.7%	82.2 ± 9.2%	86.4 ± 6.3%	70.6 ± 8.2%	91.6 ± 3.9%
	Specificity	86.5 ± 6.8%	83.7 ± 5.2%	83.4 ± 7.9%	83.8 ± 6.2%	85.3 ± 5.3%	79.5 ± 7.7%	81.8 ± 8.0%	83.4 ± 8.6%	88.5 ± 2.6%

The mean and standard deviation for the tenfold cross-validation are shown for each score.

The evaluation metrics AUROC, PPV, NPV, sensitivity, and specificity for each model are shown. Two criteria for labeling of data, a thyroid test criterion and a prescription criterion, were devised. Inst. comb. 1 represents thyroid dysfunction group data from both Wakayama Medical University and Gunma University, and a control group data from Hidaka Hospital, Inst. comb. 2 represents thyroid dysfunction group data from Wakayama Medical University and a control group data from Hidaka Hospital, and Inst. comb. 3 represents thyroid dysfunction group data from Gunma University and a control group data from Hidaka Hospital. Feature set 1 is the full set of features available in the four hospitals, and Feature set 2 is limited to five routine tests that are mandatory for Japanese national special health check-ups. Four typical machine-learning algorithms for structured data, gradient boosting decision trees, support vector machines and neural networks used in related studies, as well as logistic regression, were examined.

The results of the comparisons of different labeling criteria are shown in Nos. I and II of Table 3. When the prescription criterion was applied as the labeling criterion, the accuracy of the hyperthyroidism classification model achieved an AUROC of 93.0%, and that of the hypothyroidism classification model achieved an AUROC of 92.1%. The hyperthyroidism model trained on the thyroid function test criterion data achieved superior performance, while the hypothyroidism model trained on the prescription criterion data achieved superior performance. The results of the comparison of models built on different institution combinations is shown in Nos. I, III, and IV of Table 3, and the highest performance was obtained when institution combination 1 was used as the training set.

Among the four machine-learning algorithms used in this study, including the GBDT, the SVM, logistic regression, and the ANN, the highest performance was obtained when the GBDT method was applied, as shown in Nos. I, V, VI, and VII of Table 3. After comparing the performance of different feature sets, as shown in Nos. I and VIII of Table 3, when Feature set 2 was applied, the accuracy of the hyperthyroidism classification model was reduced, with an AUROC of 85.5%, and the performance of the hypothyroidism classification model was reduced, with an AUROC of 82.5%. Compared to Feature set 1, which is composed of the full set of features available in the four hospitals, the performance of the model constructed on Feature set 2 limited to most basic routine tests had lower accuracy, but the accuracies for hyperthyroidism (AUROC = 85.5%) and hypothyroidism (AUROC = 82.5%) were considered sufficient for rapid screening of overlooked patients.

The model of No. I in Table 3 was evaluated using the external dataset from Kuma Hospital, as shown in No. IX of Table 3. High classification performance was achieved using the external data: AUROC = 97.2%, sensitivity = 90.0%, and specificity = 93.7% for the hyperthyroidism classification model and AUROC = 94.0%, sensitivity = 91.6%, and specificity = 88.5% for the hypothyroidism classification model.

### Feature importance

**Fig. 1:**
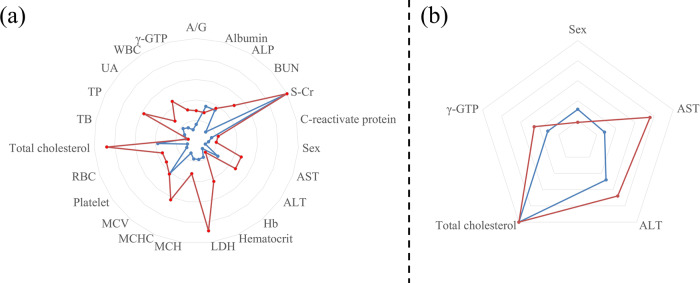
Comparison of feature importance between hyperthyroidism and hypothyroidism classification models. The blue line shows the feature importance of the hyperthyroidism classification model, while the red line shows that of the hypothyroidism classification model. **a** The five most important features in the hyperthyroidism model using Feature set 1 were S-Cr, MCV, total cholesterol, ALP, and albumin. The five most important features in the hypothyroidism model using Feature set 1 were S-Cr, LDH, total cholesterol, MCHC, and TP. **b** Among the five laboratory tests used as features, total cholesterol exhibited the highest feature importance in both the hyperthyroidism and hypothyroidism models. The second and third most important features were ALT and sex in the hyperthyroidism model, while those in the hypothyroidism model were AST and ALT. AST denotes aminotransferase, ALT: alanine aminotransferase, γ-GTP: gamma-glutamyl transpeptidase, RBC: red blood cell count, S-Cr: serum creatinine, ALP: alkaline phosphatase, UA: uric acid, LDH: lactic acid dehydr.ogenase, TP: total protein, BUN: blood urea nitrogen, A/G: albumin/globulin ratio, TB: total bilirubin, WBC: white blood count, Hb: hemoglobin, MCV: mean corpuscular volume, MCH: mean corpuscular hemoglobin, and MCHC: mean corpuscular hemoglobin concentration.

The feature importance of each model was examined using Feature set 1. The blue line of Fig. 1(a) shows the feature importance of the hyperthyroidism classification model, while the red line shows that of the hypothyroidism classification model. The five most important features in the hyperthyroidism model were S-Cr, MCV, total cholesterol, ALP, and albumin. The five most important features in the hypothyroidism model were S-Cr, LDH, total cholesterol, MCHC, and TP.

Figure 1(b) shows the feature importance when applying Feature set 2. Among the five laboratory tests used as features, total cholesterol exhibited the highest feature importance in both the hyperthyroidism and hypothyroidism models. The second and third most important features were ALT and sex in the hyperthyroidism model, while those in the hypothyroidism model were AST and ALT.

### Negative label setting

Unlike the present study, previous studies have had a drawback in that they did not consider crosstalk in the data labeling process. For hyperthyroidism classification in this study, the hyperthyroidism group was used as a positive label, and both the control and hypothyroidism groups were negatively labeled. For the hypothyroidism classification in this study, the hypothyroidism group was used as a positive label, whereas both the control and hyperthyroidism groups were negatively labeled (referred to as “crosstalk account”). On the other hand, related studies^16,17^ performed classification by setting thyroid dysfunction patients (with hyperthyroidism or hypothyroidism) as a positive label and only the control group as a negative label (referred to as “crosstalk nonaccount”). Therefore, we evaluated the performance of the models with similar settings as these studies. When only the control group was labeled negative in both the training set and the validation set, a high classification performance, with AUROCs of 98.0% and 95.7%, was achieved in the classification of overt hyperthyroidism and overt hypothyroidism, respectively, as shown in Column A-1 of Table 4. However, as shown in Column A-2 of Table 4, when both the control group and the hypothyroidism group were labeled negative in the validation set of overt hyperthyroidism and when both the control group and the hyperthyroidism group were labeled negative in the validation set of overt hypothyroidism, the classification performance was reduced, with AUROCs of 91.3% and 81.4%, respectively. The classification performance dropped greatly in the models in which crosstalk was not considered during the negative labeling process.

**Table 4:** Evaluation results obtained without considering crosstalk.

No.		A-1	A-2
Training	Positive label criterion	Thyroid function test criterion	
	Negative label setting	Crosstalk nonaccount	
Validation	Positive label criterion	Thyroid function test criterion	
	Negative label setting	Crosstalk nonaccount	Crosstalk account
Hyperthyroidism	AUROC	98.0 ± 2.2%	91.3 ± 2.3%
Hypothyroidism	AUROC	95.7 ± 3.1%	81.4 ± 4.4%

The mean and standard deviation for the tenfold cross-validation are shown for the AUROC scores. “Crosstalk account” represents the negative label setting, where both the control group and the hypothyroidism group were labeled negative in the hyperthyroidism group and both the control group and the hyperthyroidism group were labeled negative in the hypothyroidism group. “Crosstalk nonaccount” represents the negative label setting where only the control group was labeled negative.

## Discussion

In this study, we aimed to develop a machine-learning method for rapid screening of overlooked patients with hyperthyroidism and hypothyroidism who would greatly benefit from prompt medical treatment. We evaluated the diagnostic performances of machine-learning methods with routine laboratory tests as the input variables to detect the presence of thyroid dysfunction, including hyperthyroidism and hypothyroidism. High accuracy was achieved in the discrimination of evident hyperthyroidism and hypothyroidism.

### Feature importance

The correlation of routine laboratory test parameters such as S-Cr and ALP measurements with thyroid dysfunction has been noted in many previous studies. According to studies on the relationship between thyroid dysfunction and liver function^25,26^, a correlation was confirmed between increased ALP and hyperthyroidism, as the ALP value was higher when bone metabolism increased in Graves’ disease, which is a typical disorder of hyperthyroidism. Sönmez et al.^27^ examined data from 433 patients and reported that S-Cr in the hyperthyroidism group was lower than that in the euthyroid group. TSH and S-Cr were also reported to have a negative correlation with overt hypothyroidism^28^. Dorgalaleh et al.^29^ suggested that thyroid dysfunction directly affects most blood values, and health professionals must pay attention to such effects. The correlation between hypothyroidism and hyperuricemia has also been confirmed by multiple studies^30,31^.

### Comparison with related studies

Several previous studies revealed promising results of the use of machine-learning approaches for predicting thyroid dysfunction^16,17^.

Similar to the present study, Aoki et al.^17^ used pattern recognition methods such as neural networks to predict the likelihood of thyroid dysfunction from a set of routine test parameters such as ALP, S-Cr, and total cholesterol. Their results suggested that most patients with overt thyroid dysfunction could be screened by using a set of routine clinical data without measuring thyroid hormone levels. An accuracy rate of 91.3% was reported in the hyperthyroidism classification model, and an accuracy rate of 90.0% was reported in the hypothyroidism classification model. Their results suggested that there is a high correlation between a set of routine laboratory tests and thyroid dysfunction. However, the model verification of these studies used the leave-one-out method instead of cross-validation and used the correct rate as the indicator instead of the AUROC. Thus, the model evaluation was considered insufficient.

### Institution combination

The combination of institutions, Wakayama Medical University Hospital, Gunma University Hospital, and Kuma Hospital, was conducted to achieve better generalization for the machine-learning model. It was confirmed that the classification accuracy was improved by combining the dataset of two institutions (Wakayama and Gunma). Additionally, by combining datasets from different institutions, we examined whether there was bias among these institutions. According to the results from the dataset of Kuma Hospital, which was conducted as external validation in this study, no bias was observed between the institutions.

Kuma Hospital specializes in thyroid diseases; hence, its dataset is considered to have less noise than those of other general hospitals, such as Wakayama Hospital and Gunma Hospital, for which the dataset required more data cleaning.

### Regarding comparison with physician performance

When developing machine-learning-based diagnosis systems, it is usual to compare the developed systems to physician performance as a baseline. First, we confirm that the aim of this study is to screen overlooked patients with thyroid dysfunction. For this purpose, only routine laboratory tests were used to predict thyroid dysfunction diseases. On the other hand, according to the diagnostic guidelines of thyroid dysfunction, physicians start with interviews and make a definitive diagnosis by measuring TSH and FT4. In addition, there are no guidelines for diagnosing hypothyroidism from routine laboratory tests, which means that no physicians are trained to diagnose hypothyroidism from routine laboratory tests. Therefore, a diagnostic accuracy comparison with physician performance was not conducted in this study.

On the other hand, further validation such as clinical trials is required prior to the use of our method in clinical practice. We need to conduct a clinical evaluation of our method by prospectively identifying individuals suspected to have thyroid dysfunction disease and those without thyroid dysfunction, and comparing the performance of machine-learning methods with the performance of physicians who use conventional diagnostic techniques at diagnosing thyroid disorders.

### Findings on hypothyroidism classification

In the current study, the hypothyroidism classification models exhibited lower performance than the hyperthyroidism classification models. This result is attributed to differences in the respective serum hormones and underlying molecular mechanisms^32^. The various nonspecific symptoms of hypothyroidism may not manifest simultaneously, resulting in a subclinical rate higher than that of hyperthyroidism. In addition, patients with hypothyroidism, such as those with Hashimoto’s thyroiditis, are dependent upon long-term levothyroxine treatment, which may affect the manifestations of routine laboratory findings.

### Training data labeling criteria

Two labeling criteria, thyroid function test criteria and prescription criteria, were designed and compared in the present study. In the results of this study, the models trained with the thyroid function test criteria exhibited better performance. However, the model may benefit from a larger dataset that combines two different labeling criteria. Therefore, we further trained the new model on the dataset collected using both criteria and evaluated the classification performance. As a result, the model trained on both datasets exhibited an AUROC of 94.3% for hyperthyroidism and 92.2% for hypothyroidism. This result indicates that a combination of both thyroid function test criteria and prescription criteria may be effective in enhancing the classification ability of the model.

## Conclusion

This study evaluated a screening method to discriminate hyperthyroidism and hypothyroidism from EMRs or routine laboratory finding data from health checkups using a machine-learning method with the aim of preventing missed diagnosis of thyroid dysfunction. This is a versatile new screening method that was successfully developed from a machine-learning model method to discriminate patients with hyperthyroidism and those with hypothyroidism using 23 features. High accuracy was achieved in the discrimination of evident hyperthyroidism or hypothyroidism, and these features can be useful for individuals who are not thyroid disease specialists.

If thyroid dysfunction is screened using our method in health care facilities, including hospitals and health checkup facilities, prompt and accurate diagnostic support can be provided with the requirement of only routine laboratory tests. A future work is to conduct clinical trials that is required prior to the use of our method in clinical practice.

## Supplementary information

Reporting Summary

## Data Availability

Each institution owns the data of its own institution, and restrictions apply to the availability of these data, which were used under license for the current study and therefore are not publicly available. Data are, however, available from the authors upon reasonable request and with permission from each institute. Source data for Fig. 1 is available as the excel file “Figure1.xlsx” at our GitHub site: https://github.com/CosmicAITS/AITS1.

## Code availability

The code developed in this project, a simulation dataset, and instructions (README file) on how to use the program are available at our GitHub site: https://github.com/CosmicAITS/AITS1, and archived in Zenodo^33^.
